# Apolipoprotein D facilitates rabies virus propagation by interacting with G protein and upregulating cholesterol

**DOI:** 10.3389/fimmu.2024.1392804

**Published:** 2024-05-28

**Authors:** Hongyan Zhang, Xingxue Liang, Duoduo Li, Chuanliang Zhang, Wenfeng Wang, Rongze Tang, Hongyun Zhang, Abraha Bahlbi Kiflu, Cheng Liu, Jingjing Liang, Xiaoning Li, Ting Rong Luo

**Affiliations:** ^1^ State Key Laboratory for Conservation and Utilization of Subtropical Agro-Bioresources, Guangxi University, Nanning, China; ^2^ College of Animal Sciences and Veterinary Medicine, Guangxi University, Nanning, China; ^3^ Guangxi Key Laboratory of Animal Breeding, Disease Control and Prevention, Guangxi University, Nanning, China; ^4^ Guangxi Zhuang Autonomous Region Engineering Research Center of Veterinary Biologics, Guangxi University, Nanning, China

**Keywords:** rabies virus, apolipoprotein D, glycoprotein, cholesterol, viral replication

## Abstract

Rabies virus (RABV) causes a fatal neurological disease, consisting of unsegmented negative-strand RNA, which encodes five structural proteins (3′-N-P-M-G-L-5′). Apolipoprotein D (ApoD), a lipocalin, is upregulated in the nervous system after injury or pathological changes. Few studies have focused on the role of ApoD during virus infection so far. This study demonstrated that ApoD is upregulated in the mouse brain (*in vivo*) and C8-D1A cells (*in vitro*) after RABV infection. By upregulating ApoD expression in C8-D1A cells, we found that ApoD facilitated RABV replication. Additionally, Co-immunoprecipitation demonstrated that ApoD interacted with RABV glycoprotein (G protein). The interaction could promote RABV replication by upregulating the cholesterol level. These findings revealed a novel role of ApoD in promoting RABV replication and provided a potential therapeutic target for rabies.

## Introduction

Rabies virus (RABV), a typical neurotropic virus, causes a fatal zoonosis with an almost 100% mortality rate (1). RABV infects the central nervous system (CNS) that causes approximately 60,000 deaths each year worldwide (2). Vaccination and immunoglobulin are effective in mitigating and preventing the development of rabies post-virus exposure. However, specific drugs are not available to treat rabies (3, 4).

RABV is a single-stranded, negative-sense RNA virus and belongs to the genus *Lyssavirus* and the family *Rhabdoviridae* (5). The genome of RABV encodes the following five structural proteins: nucleoprotein (N), phosphoprotein (P), matrix protein (M), glycoprotein (G), and a large polymerase (L) (6). The viral RNA is encapsulated by N to form a helical nucleocapsid. The nucleocapsid along with P and L forms the ribonucleoprotein that constitutes the core of the bullet-shaped virion and the active viral unit for viral replication (6, 7). RABV G protein, which is the sole virion surface protein, plays a crucial role in viral attachment and fusion with target cell membranes. Viral budding is a complex process. Previous studies have demonstrated that the RABV M lattice is involved in inducing membrane bending for budding site formation. G protein supports this process by facilitating the formation of the M lattice, promoting viral budding (8, 9). However, the role of other cellular molecules in RABV budding has not been elucidated.

Apolipoprotein D (ApoD), a highly conserved glycoprotein, is a member of the transporter superfamily. Previous studies have reported that ApoD, a lipocalin, is involved in lipid metabolism and neuroprotective functions (10). ApoD was first detected in 1963 as a distinct component of the human plasma lipoprotein system (11) and is bound to plasma high-density lipoprotein (12). Additionally, ApoD is composed of 169 residues, including a 20–amino acid secretion signal peptide with two glycosylation sites (asparagine residues 45 and 78). The molecular weight of the mature protein varies from 20 kDa to 32 kDa (13, 14). Several potential ligands exhibiting diverse structures and functions have been identified in ApoD. ApoD is considered a multi-ligand, multi-function protein owing to the apparent heterogeneity and widespread tissue distribution of the ligands and is involved in lipid trafficking, food intake, inflammation, antioxidative response, and development (15). Several studies have confirmed that ApoD exerts neuroprotective effects against different neurodegeneration-inducing factors, such as oxidative stress, inflammatory stress, and excitotoxicity (16, 17). The neuroprotective effect of ApoD is associated with its anti-inflammatory properties and regulatory effects on neuronal cholesterol distribution and the levels of excitotoxicity-related proteins.

The upregulation of ApoD in the aging brain (18), as well as in multiple neurological conditions, suggests that ApoD is critical for neuronal maintenance and protection against injury. Rabies is an acute CNS disease with an almost 100% mortality rate. This study aimed to examine the neuroprotective mechanisms of ApoD during RABV infection. Here, we attempt to explore the interaction between ApoD and RABV. The findings of this study will improve our understanding of the functional properties of ApoD and highlight its potential as a novel therapeutic target for rabies. Based on these findings, novel therapeutic strategies may be developed for rabies, a fatal disease.

## Materials and methods

### Viruses and animals

Four RABV strains (rRC-HL, GX074, CVS-24, and CVS-11) were used in this study. The rRC-HL strain was rescued from an infectious complementary DNA (cDNA) clone pRC-HL (kindly provided by Professor Minamoto, Gifu University, Japan) based on the fixed strain RC-HL used as a vaccine for animals in Japan (19, 20). GX074 is a street RABV strain isolated from a healthy-looking dog brain in Debao County, Baise City, Guangxi Province of southern China (21, 22). CVS-24 is a mouse-adapted challenge standard RABV strain, whereas CVS-11 is a laboratory-fixed RABV strain.

All animal experiments in this study were performed in the P3 biosafety laboratory and conducted according to the ethical review of laboratory animal welfare of the People’s Republic of China (National Standard GB/T35892–2018). The animal experiments were approved by the Animal Experiment Committee of Guangxi University (approval number GXU2019–021). Male and female Kunming mice (purchased from Guangxi Medical University, Nanning, China) aged 4 weeks were intracerebrally inoculated with 30 µl of Dulbecco’s Modified Eagle Medium (DMEM) (mock) or 30 µL of DMEM containing 1,000 fluorescent focus units (FFU) of RABV. On 4 and 7 days post-infection (dpi), the mice were euthanized. The mouse brains were collected to examine the mRNA and protein levels using quantitative real-time polymerase chain reaction (qRT-PCR) and Western blotting analyses, respectively.

### Cells and plasmids

The HEK (human embryonic kidney)–293T, BSR/T7-9 (cloned from BHK-21 cells, derived from baby hamster kidney), N2A (mouse neuroblastoma), and C8-D1A cells (mouse astrocytes) were cultured in DMEM supplemented with 10% fetal bovine serum (Biological Industries) at 37°C in a humidified 5% CO_2_ incubator. The FLAG-tagged RABV G protein–encoding genes (G^-FLAG^ genes) derived from rRC-HL, GX074, and CVS-11 strains were cloned into the PCAGGS vector (pC-rRC-HL-G^-FLAG^, pC-GX074-G^-FLAG^, and pC-CVS-11-G^-FLAG^, respectively) for expression in eukaryotic cells. The FLAG-tagged RABV N protein-encoding genes (N^-FLAG^ genes), P protein-encoding genes (P^-FLAG^ genes), and M protein-encoding genes (M^-FLAG^ genes) derived from rRC-HL, GX074, and CVS-11 strains were cloned into the pcDNA3.0 vector (pcDNA3.0-N^-FLAG^, pcDNA3.0-P^-FLAG^, and pcDNA3.0-M^-FLAG^, respectively) for expression in eukaryotic cells. Additionally, the MYC-tagged ApoD (ApoD^-MYC^) (*Mus musculus*) was cloned into the pcDNA3.0 vector (pcDNA3.0-ApoD^-MYC^).

### Co-immunoprecipitation assay

HEK-293T cells were co-transfected with the mammalian expression vector pcDNA3.0-ApoD^-MYC^ (for ApoD expression) and/or PCAGGS-G^-FLAG^ (pC-rRC-HL-G^-FLAG^ and pC-GX074-G^-FLAG^) (for RABV G protein expression). At 24 h post-transfection (hpt), the cells were washed with cold phosphate-buffered saline (PBS) and lysed with NP-40 lysis buffer containing an anti-protease cocktail (100× protease inhibitor cocktail) for 40 min at 4°C. The cell lysates were centrifuged at 12,000 *g* and 4°C for 10 min. The supernatant was transferred to a new tube and incubated with mouse anti-FLAG (Abmart, Shanghai, China, M20008, 1:100) or rabbit anti-MYC (ABclonal, Wuhan, China, AE070, 1:500) monoclonal antibodies for 8 h. Next, the samples were incubated with the protein A/G agarose (Beyotime, Shanghai, China, P2055) for 8 h at 4°C with rotation. The agarose beads were washed five times with cold PBS, and the bound proteins were examined using Western blotting analysis.

### Glutathione S transferase pulldown assay

Glutathione S transferase (GST) or GST-tagged ApoD (ApoD^-GST^)–fused proteins were expressed in BL-21 cells and subsequently purified and conjugated into glutathione (GSH) beads (Solarbio, Beijing, China, P2020) at 4°C for 12 h with continuous rotation. The HEK-293T cell extracts were incubated with the GSH beads at 4°C for 12 h with continuous rotation. The protein complexes were pulled down with GSH beads and subjected to Western blotting analysis with the mouse anti-FLAG (Abmart, Shanghai, China, M20008, 1:5,000) and anti-GST monoclonal antibodies (Abmart, Shanghai, China, M20007, 1:5,000).

### Confocal microscopy

HEK-293T cells were seeded on coverslips and transfected with pC-GX074-G^-FLAG^ and pcDNA-ApoD^-MYC^ plasmids. At 24 hpt, the cells were fixed with methanol and acetone in a 1:1 ratio, probed with anti-FLAG tag and anti-MYC tag antibodies, and stained with 4′,6-diamidino-2-phenylindole. Protein localization was evaluated using a confocal microscope.

### Total RNA extraction and qRT-PCR analysis

Total RNA was extracted using the RNA isolation kit (Vazyme, Nanjing, China, RC112–01), following the manufacturer’s instructions. The quality and quantity of total RNA were evaluated using a Nanodrop 1000 spectrophotometer (Thermo, USA). qRT-PCR analysis was performed using the SYBR Green method as previously described (20). Briefly, 1 μg of total RNA (template) was subjected to first-strand cDNA synthesis using the Hiscript II Q RT SuperMix for qRT-PCR (+gDNA wiper), following the manufacturer’s instructions. A LightCycler 96 PCR detection system (Roche Diagnostics Ltd.) was used for quantitative assessment of the mRNA levels of genes encoding ApoD and RABV N, P, M, and G under the standard cycling conditions. The β-actin–encoding gene was used as a control in all reactions. The primer sequences used in qRT-PCR analysis are provided in **Supplementary Table 1**.

### Western blotting analysis

Western blotting analysis was performed as previously described (20). C8-D1A, BSR/T7-9, and N2A cells were lysed using radioimmunoprecipitation assay (RIPA) lysis buffer containing protease inhibitors. The lysate was subjected to sodium dodecyl sulfate-polyacrylamide gel electrophoresis using a 12% gel. The resolved proteins were transferred onto a polyvinylidene difluoride (PVDF) membrane (Millipore, Billerica, MA). The PVDF membrane was blocked with 5% skim milk powder in 1× Tris-buffered saline containing Tween-20 (TBST) for 2 h at room temperature. Next, the membrane was incubated with primary antibodies at 4°C overnight. After washing five times with 1× TBST, the membrane was incubated with the corresponding secondary antibody for 2 h at 37°C. The membrane was washed five times with 1× TBST, and the immunoreactive signals were visualized using a 5-bromo-4-chloro-3-indolyl-phosphate/nitro blue tetrazolium kit (Beyotime Ltd, China). Immunoreactivity was quantified using densitometric analysis with an Odyssey scanner (Li-Cor, Lincoln, NE, USA).

### Antibodies

Specific antibodies were used for the Western blotting analysis. Mouse anti-ApoD monoclonal antibody was purchased from Santa Cruz Biotechnology Co., Ltd. (CA, USA, sc-166612, 1:1,000); mouse anti-RABVM protein monoclonal antibody was obtained from CUSABIO™Co., Ltd. (WuHan, China, 1–202AA, 1:2,000); and mouse anti-RABV N protein monoclonal antibody was purchased from Hangzhou Dayao Biotechology™ Co., Ltd. (Hangzhou, China, Ab-0056, 1:20,000). Mouse anti–β-actin monoclonal antibody was purchased from Beijing ComWin Biotech™ Co., Ltd. (Beijing, China, cw0096A, 1:1,000). Mouse anti-RABV P and G protein monoclonal antibodies were kindly provided by Dr. Minamoto Nobuyuki (Gifu University, Japan).

### Virus infection and titration

C8-D1A cells and N2A cells were infected with rRC-HL at an indicated multiplicity of infection (MOI) for 2 h at 37°C. The cell culture supernatants and cells were collected at 12, 24, and 48 h post-infection (hpi) for virus titration. Viral titers were determined using the indirect immunofluorescence assay (IFA) as previously described (23).

### Cell viability assay

Cell viability was analyzed using the cell counting kit–8 (CCK-8) method, following the manufacturer’s instructions. Briefly, the C8-D1A cells were seeded in 96-well microplates with six replicates and pre-treated with or without cholesterol (Sigma, USA) at 5% CO_2_ and 37°C for 24 h. Next, the cells were incubated with the CCK-8 reagent at 37°C for 1 h. Finally, the absorbance of the reaction mixture was determined using a Microplate Reader (Tecan Infinite 200Pro, Switzerland). The data were analyzed using GraphPad software.

### Cellular cholesterol assay

To quantify the total cellular cholesterol level, C8-D1A cells were seeded in 12-well plates and transfected with empty vector, pcDNA-ApoD^-MYC^, and pC-GX074-G^-FLAG^ plasmids and co-transfected with pcDNA-ApoD^-MYC^ and pC-GX074-G^-FLAG^ plasmids, separately. After washing thrice with PBS, the cells were harvested and lysed. The cholesterol level was quantified using a cholesterol quantification kit (Catalog; 40006; AAT Bioquest, USA), following the manufacturer’s instructions.

### Statistical analysis

Data were expressed as the mean ± SD (standard deviation). Significances were calculated from at least three independent experiments using GraphPad Prism (8.0.0, MA, USA) by the Student’s t-test for unpaired data or the one/two-way analysis of variance, as indicated in the figure legend. Asterisks indicate statistical significance (*P < 0.05, **P < 0.01, ***P < 0.001, and ****P < 0.0001).

## Results

### RABV infection upregulates ApoD expression

**Figure 1:**
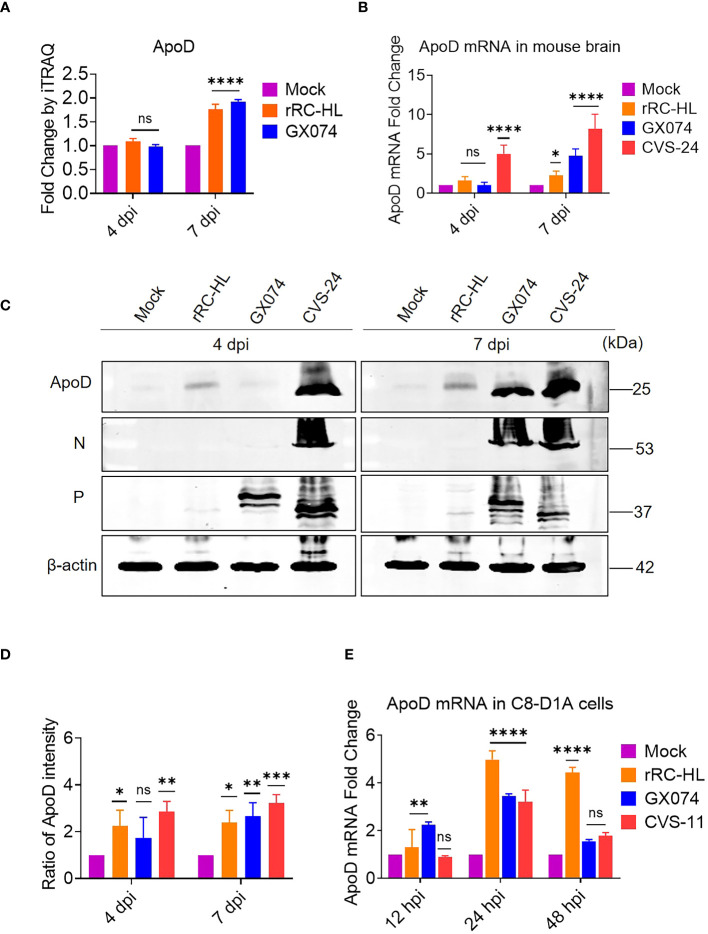
Rabies virus (RABV) infection upregulates apolipoprotein D (ApoD) expression. Kunming mice were intracerebrally inoculated with 30 μL of Dulbecco’s Modified Eagle Medium (DMEM) containing 1,000 fluorescent focus units (FFU) of three different RABV strains (rRC-HL, GX074, and CVS-24) or mock-infected with 30 μL of DMEM. At 4 and 7 days post-infection (dpi), the mouse brain was harvested for further analysis. **(A)** The ApoD protein level in the mouse brain after infection with rRC-HL and GX074 strains was quantified using iTRAQ analysis. **(B)** Total RNA of the brain tissue was extracted and subjected to quantitative real-time polymerase chain reaction (qRT-PCR) analysis to examine the mRNA level of ApoD. **(C)** The protein levels of ApoD and RABV N and P in the brain tissue of RABV-infected and mock-infected mice were examined at 4 and 7 dpi using Western blotting analysis. β-actin was used as a reference protein. **(D)** The ApoD protein level was quantified using ImageJ software. The levels of ApoD protein were standardized to those of β-actin and normalized to those in the mock-infected mouse brain. **(E)** C8-D1A cells were infected with rRC-HL at a multiplicity of infection (MOI) of 0.1 or mock-infected. At 12, 24, and 48 h post-infection (hpi), cells were collected and subjected to qRT-PCR analysis to examine the mRNA level of ApoD. Data are represented as mean ± standard deviation. Statistical differences were analyzed using two-way analysis of variance (ns, non-significant; ^*^ *P* < 0.05, ^**^ *P* < 0.01, ^***^ *P* < 0.001, and ^****^ *P* < 0.0001).

Proteomic analysis using iTRAQ revealed that ApoD expression was significantly altered at 7 dpi. In particular, the ApoD levels in rRC-HL–infected and GX074-infected mice were 1.8-fold and 1.9-fold higher, respectively, than those in mock-infected mice at 7 dpi (**Figure 1A**). The RABV-infected mouse brain was harvested at 4 and 7 dpi and homogenized. The mRNA and protein levels of ApoD were examined using qRT-PCR and Western blotting analyses, respectively. Compared with those in mock-infected mice, the brain ApoD mRNA level was five-fold higher in CVS-24–infected mice and was slightly upregulated in rRC-HL–infected and GX074-infected mice at 4 dpi. At 7 dpi, the brain ApoD mRNA level in rRC-HL–infected, GX074-infected, and CVS-24–infected mice was 2.3-fold, 4.8-fold, and 8.2-fold higher, respectively, than that in mock-infected mice (**Figure 1B**). Consistently, Western blotting analysis revealed that the brain protein level of ApoD in RABV-infected mice was markedly higher than that in mock-infected mice (**Figure 1C**). Compared with that in mock-infected mice, the brain ApoD intensity was 2.2-fold and 2.9-fold higher in rRC-HL–infected and CVS-24–infected mice, respectively, at 4 dpi and was 2.4-fold, 2.7-fold, and 3.2-fold higher in rRC-HL–infected, GX074-infected, and CVS-24–infected mice, respectively, at 7 dpi (**Figure 1D**).

To further confirm these results *in vitro*, C8-D1A cells were infected with rRC-HL, GX074, or CVS-11 strains at an MOI of 0.1. The mRNA level of ApoD was measured using qRT-PCR analysis. Compared with those in mock-infected cells, the ApoD mRNA levels were 1.3-fold and 2.4-fold higher in rRC-HL–infected and GX074-infected C8-D1A cells, respectively, at 12 hpi. However, the ApoD mRNA levels were not significantly different between CVS-11-infected at 12 hpi. The ApoD mRNA levels in rRC-HL–infected, GX074-infected, and CVS-24–infected cells were 5.0-fold, 3.5-fold, and 3.2-fold higher, respectively, than those in mock-infected cells at 24 hpi. At 48 hpi, only the ApoD mRNA level in rRC-HL–infected cells was significantly upregulated (by 4.4-fold) when compared with that in mock-infected cells (**Figure 1E**). These findings suggested that RABV infection significantly upregulates ApoD expression both *in vitro* and *in vivo*.

### ApoD overexpression promotes RABV replication in C8-D1A cells

**Figure 2:**
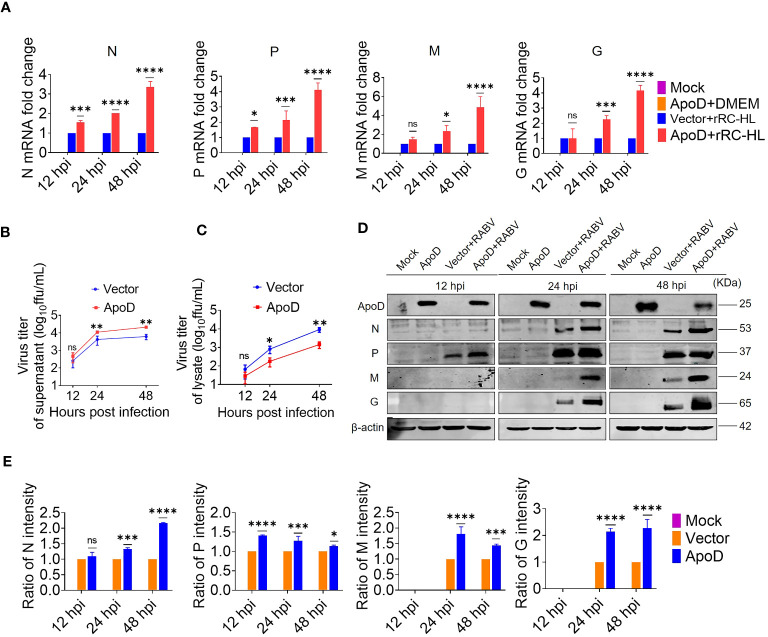
Apolipoprotein D (ApoD) overexpression promotes rabies virus (RABV) replication in C8-D1A cells. ApoD overexpression upregulated the mRNA level of RABV genes, the viral titer in the cell supernatant, and the viral protein expression level in cells. C8-D1A cells were transfected with 1 μg of the empty vector or pcDNA-ApoD^-MYC^ plasmid. At 12 h post-transfection (hpt), the cells were infected with rRC-HL at a multiplicity of infection (MOI) of 0.1. **(A)** At 12, 24, and 48 h post-infection (hpi), C8-D1A cells were lysed with buffer RL, and the mRNA levels of RABV N, P, M, and G genes were examined using quantitative real-time polymerase chain reaction (qRT-PCR) analysis. **(B, C)** At 12, 24, and 48 hpi, the culture supernatant and the lysate of C8-D1A cells were collected, and the viral titers were tested with BSR/T7-9 cells. **(D)** At 12, 24, and 48 hpi, C8-D1A cells were lysed with radioimmunoprecipitation assay buffer, and the protein expression levels of RABV N, P, M, and G were examined using Western blotting analysis. **(E)** Statistical analysis of the protein expression levels of RABV N, P, M, and G proteins was performed using ImageJ software. The expression levels of RABV N, P, M, and G proteins were standardized to those of β-actin and normalized to those of empty vector–transfected cells. Data are represented as mean ± standard deviation. Two-way analysis of variance for **(A, E)**; Student’s t-test for **(B, C)** (ns, non-significant; ^*^ *P* < 0.05, ^**^ *P* < 0.01, ^***^ *P* < 0.001, and ^****^ *P* < 0.0001).

Based on above experimental results, RABV infection could upregulate ApoD expression *in vitro* and *in vivo*. Next, the role of ApoD in RABV infection was examined. C8-D1A cells were transiently transfected with a pcDNA-ApoD^-MYC^ plasmid to overexpress ApoD. ApoD-overexpressing C8-D1A cells were infected with the rRC-HL strain. The mRNA and protein levels of N, P, M, and G genes were evaluated using qRT-PCR and Western blotting analyses, respectively. Additionally, the titer of rRC-HL strains was examined. The mRNA levels of viral genes in ApoD-overexpressing cells were significantly higher than those in empty vector–transfected cells at 12, 24, and 48 hpi. In particular, the N mRNA levels in pcDNA-ApoD^-MYC^–transfected cells were 1.5-fold, 2.0-fold, and 3.4-fold higher than those in empty vector–transfected cells at 12, 24, and 48 hpi, respectively. The P mRNA levels in pcDNA-ApoD^-MYC^–transfected cells were 1.7-fold, 2.14-fold, and 4.1-fold higher than those in empty vector–transfected cells at 12, 24, and 48 hpi, respectively. The M mRNA levels in pcDNA-ApoD^-MYC^–transfected cells were 2.3-fold and 4.9-fold higher than those in empty vector–transfected cells at 24 and 48 hpi, respectively. The G mRNA levels in pcDNA-ApoD^-MYC^–transfected cells were 2.2-fold and 4.2-fold higher than those in empty vector–transfected cells at 24 and 48 hpi, respectively (**Figure 2A**). The rRC-HL titers in the culture supernatant of C8-D1A cells transfected with pcDNA-ApoD^-MYC^ plasmid were 4.69 × 10^2^ FFU/mL, 1.11 × 10^4^ FFU/mL, and 2.13 × 10^4^ FFU/mL at 12, 24, and 48 hpi, respectively, which were significantly higher than those in empty vector–transfected cells (2.57 × 10^2^ FFU/mL, 4.25 × 10^3^ FFU/mL, and 6.17 × 10^3^ FFU/mL, respectively) (**Figure 2B**). In contrast, the intracellular rRC-HL titers in pcDNA-ApoD^-MYC^–transfected C8-D1A cells were 2.87 × 10^1^, 1.79 × 10^2^, and 1.42 × 10^3^ FFU/mL at 12, 24, and 48 hpi, respectively, which were significantly lower than those in empty vector–transfected C8-D1A cells (6.6 × 10^1^ FFU/mL, 8.12 × 10^2^ FFU/mL, and 9.16 × 10^3^ FFU/mL, respectively) (**Figure 2C**). The protein levels of rRC-HL N, P, M, and G were measured using Western blotting. The rRC-HL N protein levels in pcDNA-ApoD^-MYC^–transfected cells were 1.33-fold and 2.16-fold higher than those in empty vector–transfected cells at 24 and 48 hpi, respectively. Meanwhile, the P protein levels in pcDNA-ApoD^-MYC^–transfected cells were 1.4-fold, 1.3-fold, and 1.1-fold higher than those in empty vector–transfected cells at 12, 24, and 48 hpi, respectively. The M protein levels in pcDNA-ApoD^-MYC^–transfected cells were 1.80-fold and 1.44-fold higher than those in empty vector–transfected cells at 24 and 48 hpi, respectively. The G protein levels in pcDNA-ApoD^-MYC^–transfected cells were 2.1-fold and 2.3-fold higher than those in empty vector–transfected cells at 24 and 48 hpi, respectively (**Figures 2D, E**). Analysis of viral mRNA, proteins, and titers suggested that ApoD promotes RABV replication in C8-D1A cells.

### ApoD upregulates RABV G protein expression

**Figure 3:**
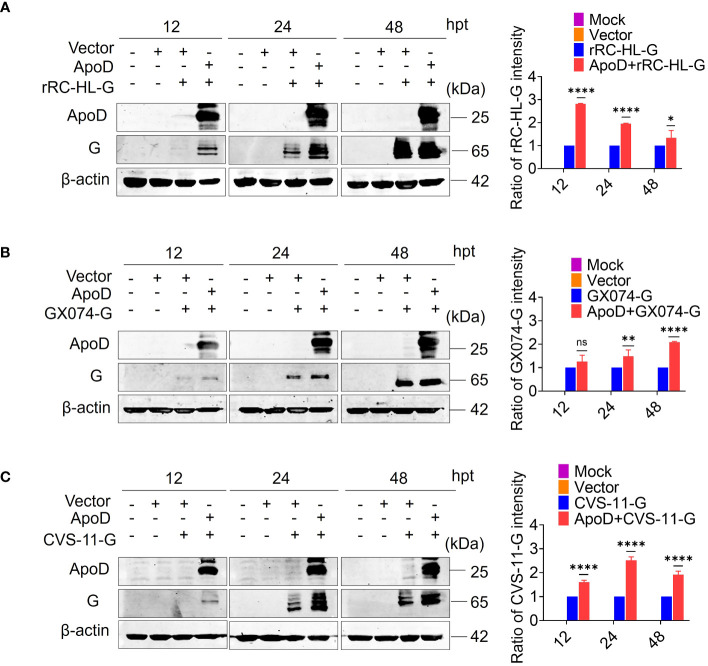
Apolipoprotein D (ApoD) upregulates rabies virus (RABV) G protein expression. **(A–C)** BSR/T7-9 cells were transfected with 1 µg of pcDNA-ApoD^-MYC^ plasmid. At 12 h post-transfection (hpt), cells were transfected with 1 µg pC-rRC-HL-G^-FLAG^, pC-GX074-G^-FLAG^, or pC-CVS-11-G^-FLAG^ plasmid. At 12, 24, and 48 h, the G genes of rRC-HL, GX074, and CVS-11 were expressed in BSR/T7-9 cells. The cell lysates were prepared using radioimmunoprecipitation assay lysis buffer. The protein expression levels of rRC-HL G **(A)**, GX074 G **(B)**, and CVS-11 G **(C)** were examined using Western blotting analysis. ImageJ software was used to quantify the G protein level. The expression levels of G protein were standardized to those of β-actin and normalized to those in cells not transfected with pcDNA-ApoD^-MYC^. Data are represented as mean ± standard deviation. Statistical differences were analyzed using two-way analysis of variance (ns, non-significant; *P < 0.05, ^**^ *P* < 0.01, and ^****^ *P* < 0.0001).

Previous experiments have suggested that ApoD could promote RABV replication. To further investigate how the ApoD impact RABV replication. The ApoD plays a role on viral mRNA or viral protein levels. BSR/T7-9 cells were transfected with 1 µg of pcDNA-ApoD^-MYC^ plasmid for 12 h, followed by transfection with 1 µg of pC-rRC-HL-G^-FLAG^, pC-GX074-G^-FLAG^, or pC-CVS-11-G^-FLAG^ plasmid. The cell lysates were prepared at 12, 24, and 48 hpt using RIPA buffer. The G protein expression level was examined using Western blotting analysis. The rRC-HL G, GX074 G, and CVS-11 G levels in pcDNA-ApoD^-MYC^–transfected cells were significantly higher than those in mock-transfected cells at 12, 24, and 48 hpt. The expression levels of rRC-HL G protein in pcDNA-ApoD^-MYC^–transfected cells were 2.81-fold, 1.95-fold, and 1.33-fold higher than those in mock-transfected cells at 12, 24, and 48 hpt, respectively (**Figure 3A**). The GX074 G expression levels in pcDNA-ApoD^-MYC^–transfected cells were 1.3-fold, 1.5-fold, and 2.1-fold higher than those in mock-transfected cells at 12, 24, and 48 hpt, respectively (**Figure 3B**). The CVS-11 G levels in pcDNA-ApoD^-MYC^–transfected cells were 1.6-fold, 2.51-fold, and 1.91-fold higher than those in mock-transfected cells at 12, 24, and 48 hpt, respectively (**Figure 3C**). Next, the effect of ApoD on the expression of other RABV proteins (N, P, and M) was examined. The experimental procedure used to evaluate RABV N, P, and M protein levels was similar to that used to evaluate RABV G protein levels. ApoD did not significantly affect the expression of RABV N, P, and M proteins (**Supplementary Figure 1**). These results suggest that ApoD regulated the expression of RABV G protein but not that of N, P, and M proteins.

### ApoD interacts with RABV G protein

**Figure 4:**
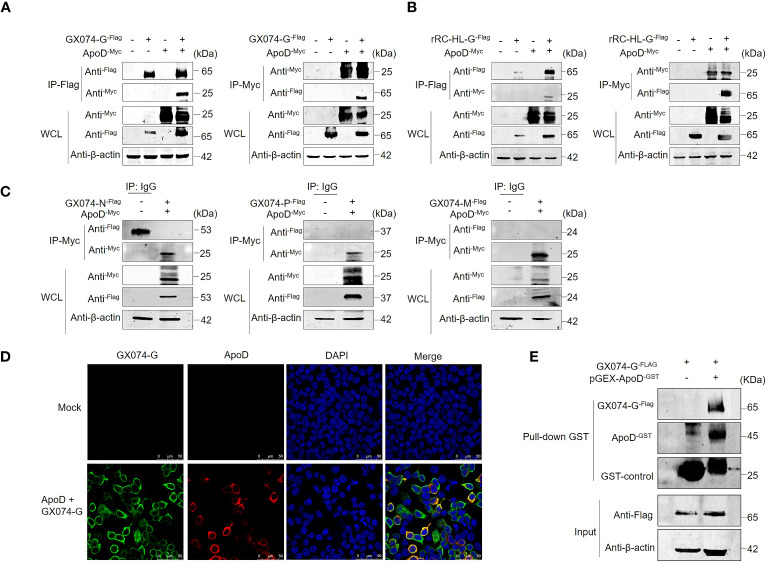
Apolipoprotein D (ApoD) interacts with rabies virus (RABV) G protein. **(A, B)** HEK-293T cells were co-transfected with pC-GX074-G^-FLAG^ and pcDNA-ApoD^-MYC^ plasmids **(A)** or pC-rRC-HL-G^-FLAG^ and pcDNA-ApoD^-MYC^ plasmids **(B)**. Cells transfected with individual plasmids served as a control. The cells were lysed with NP-40 lysis buffer and subjected to co-immunoprecipitation (Co-IP) using a monoclonal antibody (Mab) against the FLAG/MYC tag. Western blotting analysis was performed to detect the products from the immunoprecipitation and the whole-cell lysate with the relevant antibodies. **(C)** HEK-293T cells were co-transfected with pcDNA-GX074-N^-FLAG^, pcDNA-GX074-P^-FLAG^, or pcDNA-GX074-M^-FLAG^ plasmids and pcDNA-ApoD^-MYC^ plasmid. Cell extracts were used to perform immunoprecipitation with IgG or anti-MYC antibody. The products of immunoprecipitation and the whole-cell lysate were examined using Western blotting analysis with the relevant antibodies. **(D)** HEK-293T cells were co-transfected with pC-GX074-G^-FLAG^ and pcDNA-ApoD^-MYC^ plasmids or mock-transfected for 24 h, fixed with methanol and acetone at a 1:1 ratio, and stained with anti-MYC and anti-FLAG Mabs and 4′,6-diamidino-2-phenylindole (DAPI). The cells were observed under a confocal fluorescence microscope. Scale bar, 50 μm. **(E)** Glutathione S transferase (GST) pulldown assay of ApoD and GX074 G protein. The GST-ApoD–fused protein (ApoD^-GST^) and GST were purified and incubated with HEK-293T whole-cell lysate. ApoD, GX074 G protein, and GST control in the pulldown samples were detected using Western blotting analysis with specific antibodies.

Previous experiments have showed that ApoD upregulates RABV G protein expression. To further investigate the mechanism how the ApoD promotes RABV replication and to explore whether ApoD interacts with RABV G protein, ApoD and RABV G protein were subjected to co-immunoprecipitation (Co-IP) assay. HEK-293T cells were co-transfected with the pC-GX074-G^-FLAG^ and pcDNA-ApoD^-MYC^ plasmids and harvested at 24 hpt. The Co-IP assay was performed with anti-MYC or anti-FLAG antibodies. ApoD could interact with GX074 G protein (**Figure 4A**). Parallel experiments for analyzing the interaction between ApoD and rRC-HL G protein were performed. ApoD could interact with rRC-HL G protein (**Figure 4B**). To further explore the interaction of ApoD with GX074 N, P, and M proteins, pcDNA-GX074-N^-FLAG^, pcDNA-GX074-P^-FLAG^, and pcDNA-GX074-M^-FLAG^ plasmids were constructed. HEK-293T cells were co-transfected with pcDNA-GX074-N^-FLAG^/pcDNA-ApoD^-MYC^, pcDNA-GX074-P^-FLAG^/pcDNA-ApoD^-MYC^, or pcDNA-GX074-M^-FLAG^/pcDNA-ApoD^-MYC^. ApoD did not interact with N, P, and M proteins of the street strain GX074 (**Figure 4C**). In short, the results revealed that ApoD could interact with the G protein from the street strain GX074 and attenuate strain rRC-HL.

To further identify the interaction between ApoD and GX074 G protein, a protein–protein colocalization test was performed. HEK-293T cells were co-transfected with pC-GX074-G^-FLAG^ and pcDNA-ApoD^-MYC^ plasmids for 24 h. The HEK-293T cells expressing GX074-G^-FLAG^ and ApoD^-MYC^ proteins were fixed and subjected to IFA using anti-FLAG tag and anti-MYC tag antibodies. GX074-G^-FLAG^ co-localized with ApoD^-MYC^ proteins in the cytoplasm of HEK-293T cells (**Figure 4D**), further confirming the interaction between ApoD and G protein.

The GST pulldown assay was performed to verify the ApoD–G protein interaction. GST-fused ApoD (ApoD^-GST^) was expressed and purified. ApoD^-GST^ was used to fish the GX074 G protein expressed in the HEK-293T cells (transfected with pC-GX074-G^-FLAG^) using the GST pulldown assay. The result revealed that the ApoD^-GST^ could directly interact with GX074 G protein *in vitro* (**Figure 4E**).

### ApoD–G protein interaction facilitates RABV replication

**Figure 5:**
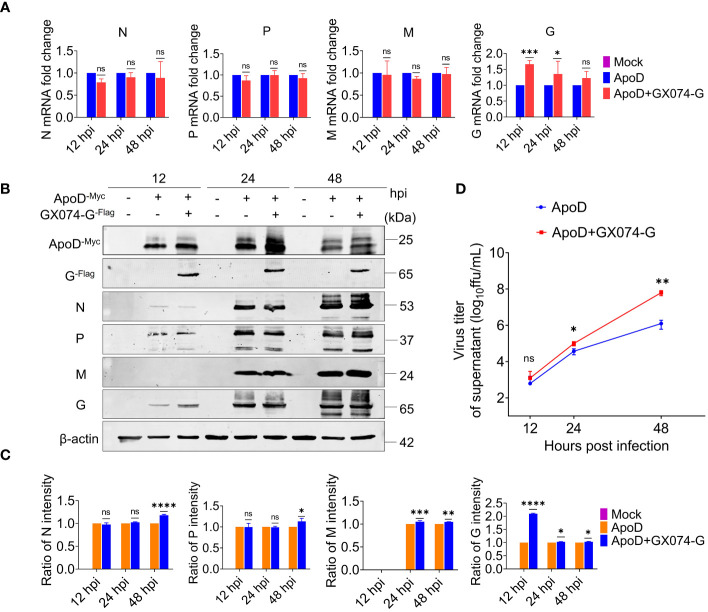
Apolipoprotein D (ApoD)–G protein interaction facilitates rabies virus (RABV) replication. N2A cells were co-transfected with pcDNA-ApoD^-MYC^ and pC-GX074-G^-FLAG^ plasmids. The control group was transfected with pcDNA-ApoD^-MYC^ plasmid alone. Next, the cells were infected with rRC-HL at a multiplicity of infection (MOI) of 0.1 at 24 h post-transfection (hpt). **(A)** At 12, 24, and 48 h post-infection (hpi), N2A cells were lysed with buffer RL. The mRNA levels of RABV N, P, M, and G genes were measured using quantitative real-time polymerase chain reaction (qRT-PCR) analysis. **(B)** At 12, 24, and 48 hpi, N2A cells were lysed with radioimmunoprecipitation assay lysis buffer. The protein expression levels of RABV N, P, M, and G were examined using Western blotting analysis. **(C)** Statistical analysis of the N, P, M, and G protein levels in the cells was performed using ImageJ software. The expression levels of N, P, M, and G proteins were standardized to those of β-actin and normalized to those in pcDNA-ApoD^-MYC^–transfected cells. **(D)** At 12, 24, and 48 hpi, the culture supernatant of N2A cells was collected. The viral titer was tested using BSR/T7-9 cells. Data are represented as mean ± standard deviation. Two-way analysis of variance for **(A, C)**; Student’s t-test for **(D)**. (ns, non-significant; ^*^ *P* < 0.05; ^**^ *P* < 0.01; ^***^ *P* < 0.001; ^****^ *P* < 0.0001).

Next, to investigate how the ApoD–G protein interaction facilitates RABV replication or the ApoD–G protein interaction influences which step of the viral cycle. N2A cells were co-transfected with pcDNA-ApoD^-MYC^ and pC-GX074-G^-FLAG^ plasmids. The control group was transfected with pcDNA-ApoD^-MYC^ plasmid alone. At 24 hpt, the N2A cells were infected with RABV rRC-HL strain at an MOI of 0.1. The N2A cells were collected and used to detect the mRNA and protein expression levels of rRC-HL genes. The mRNA levels of rRC-HL N, P, and M genes in pcDNA-ApoD^-MYC^/pcDNA-GX074-G^-FLAG^–co-transfected cells were not significantly higher than those in pcDNA-ApoD^-MYC^–transfected cells at 12, 24, and 48 hpi. Compared with those in pcDNA-ApoD^-MYC^–transfected cells, the mRNA levels of rRC-HL G gene were 1.7-fold higher at 12 hpi and 1.3-fold and 1.2-fold higher at 24 and 48 hpi, respectively, in pcDNA-ApoD^-MYC^/pcDNA-GX074-G^-FLAG^–co-transfected cells (**Figure 5A**). The expression levels of rRC-HL N, P, M, and G proteins in pcDNA-ApoD^-MYC^/pcDNA-GX074-G^-FLAG^–co-transfected cells were higher than those in pcDNA-ApoD^-MYC^–transfected cells at 12, 24, and 48 hpi. In particular, the expression levels of rRC-HL N protein in pcDNA-ApoD^-MYC^/pcDNA-GX074-G^-FLAG^–co-transfected cells were 1.18-fold higher than those in pcDNA-ApoD^-MYC^–transfected cells at 48 hpi. Meanwhile, the rRC-HL P protein expression levels in pcDNA-ApoD^-MYC^/pcDNA-GX074-G^-FLAG^–co-transfected cells were 1.13-fold higher than those in pcDNA-ApoD^-MYC^–transfected cells at 48 hpi. The rRC-HL M protein expression levels in pcDNA-ApoD^-MYC^/pcDNA-GX074-G^-FLAG^–co-transfected cells were 1.05-fold and 1.04-fold higher than those in pcDNA-ApoD^-MYC^–transfected cells at 24 and 48 hpi, respectively. The rRC-HL G protein expression levels in pcDNA-ApoD^-MYC^/pcDNA-GX074-G^-FLAG^–co-transfected cells were 2.1-fold, 1.03-fold, and 1.03-fold higher than those in pcDNA-ApoD^-MYC^–transfected cells at 12, 24, and 48 hpi, respectively (**Figures 5B, C**). Next, the rRC-HL virus titer in the culture supernatant was measured. The rRC-HL virus titers in the supernatant of N2A cells co-transfected pcDNA-ApoD^-MYC^ and pC-GX074-G^-FLAG^ plasmids were 1.24 × 10^3^ FFU/mL, 1.0 × 10^5^ FFU/mL, and 6.28 × 10^7^ FFU/mL at 12, 24, and 48 hpt, respectively, which were significantly higher than those in the supernatant of cells transfected with pcDNA-ApoD^-MYC^ plasmid (6.18 × 10^2^ FFU/mL, 3.72 × 10^4^ FFU/mL, and 1.25 × 10^6^ FFU/mL, respectively) (**Figure 5D**). Taken together, these results confirmed that ApoD–G protein interaction facilitates RABV replication.

### ApoD–G protein interaction upregulates the cholesterol level

**Figure 6:**
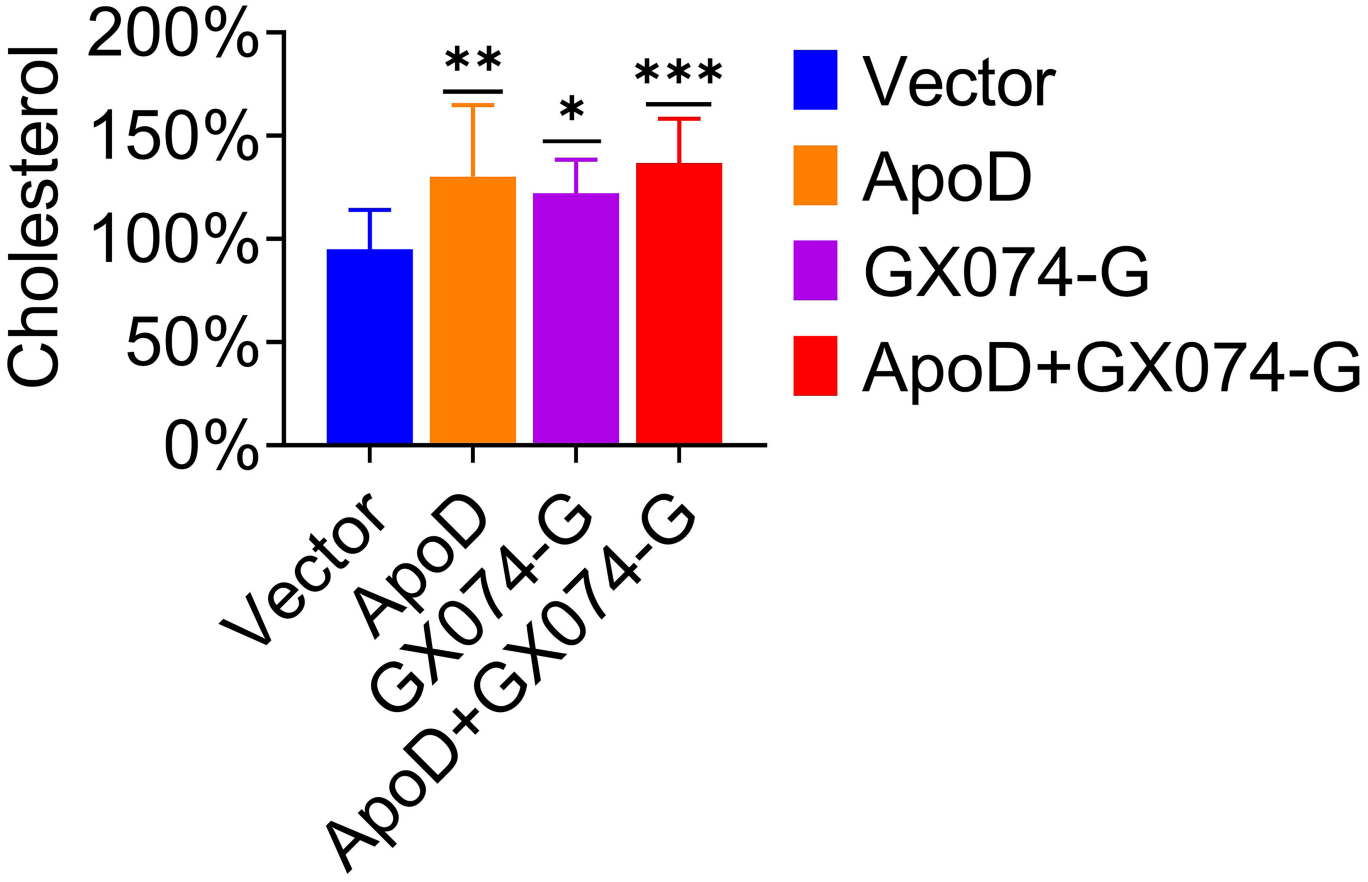
Apolipoprotein D (ApoD)–G protein interaction upregulates cholesterol level. C8-D1A cells were transfected with empty vector, pcDNA-ApoD^-MYC^, pC-GX074-G^-FLAG^ plasmid, and co-transfected with pcDNA-ApoD^-MYC^ and pC-GX074-G^-FLAG^ plasmids, separately. These transfected cells were harvested at 48 h post-transfection (hpt) to detect the cholesterol level using a cholesterol kit (n = 6). Statistical differences were analyzed using one-way analysis of variance (ns, non-significant; ^*^ *P* < 0.05, ^**^ *P* < 0.01, and ^***^ *P* < 0.001).

Based on the above experimental results, we hypothesized that ApoD–G protein interaction is correlated with cholesterol. Thus, the cholesterol level in C8-D1A cells was measured using the Amplex Red cholesterol assay. As shown in **Figure 6**, the cholesterol level in pcDNA-ApoD^-MYC^–transfected cells were significantly higher (36% higher) than those in empty vector–transfected cells. Transfection with GX074-G^-FLAG^ plasmid increased the cholesterol level by 28%, whereas co-transfection with pcDNA-ApoD^-MYC^ and pC-GX074-G^-FLAG^ increased the cholesterol level by 44%. This indicated that ApoD–G protein interaction could further upregulate the cholesterol level and that cholesterol upregulation might promote virus replication.

### Cholesterol promotes RABV replication

**Figure 7:**
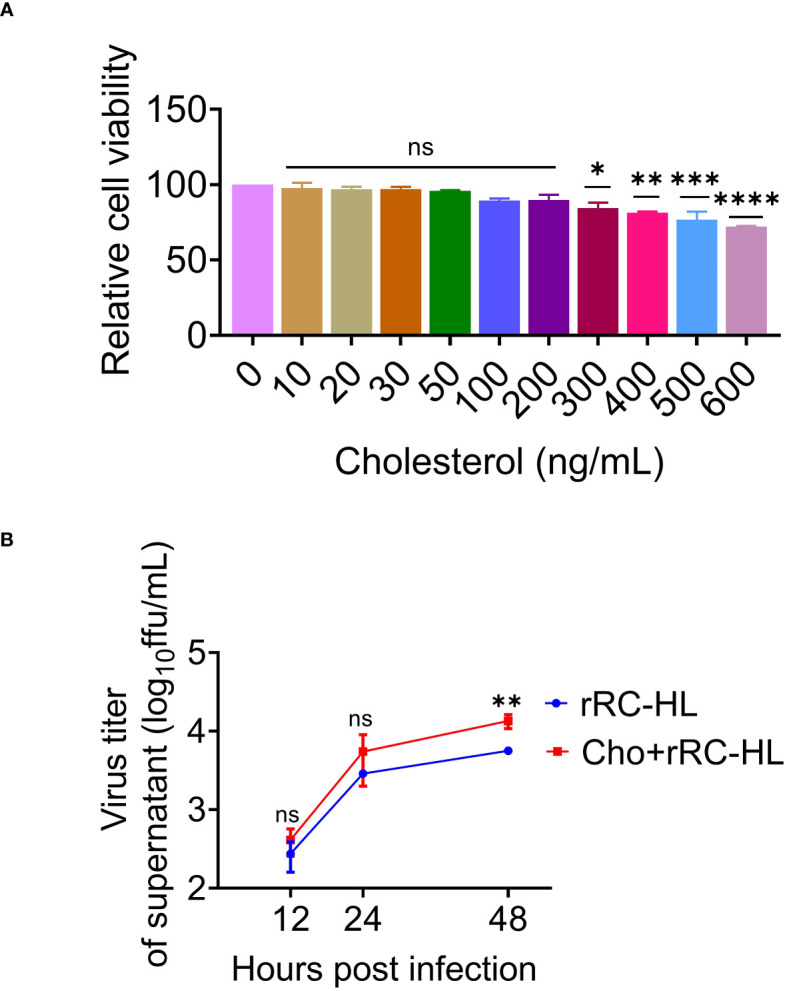
Cholesterol promotes rabies virus (RABV) replication. **(A)** C8-D1A cells were treated with different concentrations of cholesterol for 24 h. The effect of cholesterol on cell viability was determined using the cell counting kit–8 (CCK-8) assay. **(B)** C8-D1A cells were treated with a fresh medium containing water-soluble cholesterol at 37°C for 4 h. The control group was treated with fresh medium alone. The cells were then infected with rRC-HL at a multiplicity of infection (MOI) of 0.1. At 12, 24, and 48 h post-infection (hpi), the culture supernatant of C8-D1A cells was collected to examine the viral titer using BSR/T7-9 cells. Data are represented as mean ± standard deviation. One-way analysis of variance for **(A)**; Student’s t-test for **(B)** (ns, non-significant; ^*^ *P* < 0.05, ^**^ *P* < 0.01, ***P <0.001 and ****P < 0.0001).

Cholesterol is the main component of cell membrane lipid rafts, which are the specific membrane microdomains required for the entry, biosynthesis, assembly, and budding of various viruses. Viperin was reported to inhibit RABV budding by downregulating the levels of cholesterol and sphingomyelin (20). To further determine the role of cholesterol in RABV replication, a type of water-soluble cholesterol was used to examine the effect of cholesterol on RABV replication. The effect of cholesterol on cell viability was examined using the CCK-8 assay to determine the toxic concentration of cholesterol for C8-D1A cells. Cholesterol exerted toxic effects on C8-D1A cells at concentrations of ≥ 300 ng/mL (**Figure 7A**).

To confirm the effect of cholesterol on RABV rRC-HL replication, C8-D1A cells were treated with a fresh medium containing water-soluble cholesterol (200 ng/mL) at 37°C for 4 h, followed by infection with rRC-HL at an MOI of 0.1. The RABV rRC-HL titers in the culture supernatant of water-soluble cholesterol-treated cells were 4.12 × 10^2^ FFU/mL, 5.5 × 10^3^ FFU/mL, and 1.35 × 10^4^ FFU/mL at 12, 24, and 48 hpi respectively, which were significantly higher than those in untreated cells (2.75 × 10^2^ FFU/mL, 2.89 × 10^3^ FFU/mL, and 5.63 × 10^3^ FFU/mL, respectively) (**Figure 7B**). This confirmed that cholesterol promotes RABV replication.

## Discussion

RABV, a highly neurotropic pathogen, causes fatal encephalitis in warm-blooded mammals, including humans. RABV transmitted to humans and animals by biting or scratching from the wound site to the CNS. At the CNS, RABV establishes a productive infection and replication without interference by the peripheral immune system (4, 24). The replication of RABV in the nervous system, especially in the CNS, can be attributed to a complex process involving receptor-mediated absorption, membrane fusion, entry, transcription, synthesis, and assembly of proteins and budding. The G protein mediates the absorption and membrane fusion process of RABV. Viral replication is initiated after RABV is internalized into host cells via clathrin-mediated endocytosis (4, 25).

Apolipoprotein is a key regulator of lipid transport and lipoprotein metabolism. ApoD occurs in the macromolecular complex with lecithin-cholesterol acetyltransferase and mediates the transport and binding of bilin (12). ApoD is involved in a lot of biological processes and also closely associated with many neurological diseases. Limited studies have reported the effect of ApoD on viral replication, especially neurotropic viral replication. ApoD gene was previously reported to be upregulated in the CNS of mice infected with encephalitis-associated viruses, such as Japanese encephalitis virus (26), herpes simplex type-1 virus (27), and RABV (28).

Lipid rafts, which are an important membrane lipid domain, have been considered an active region for host-virus interactions and play an important role during the viral lifecycle (29–31). Several enveloped viruses use cholesterol in lipid rafts for infection (32, 33). Cholesterol, an important component of the cell membrane, is critical for maintaining cell viability, signaling, and physiology (34). Previous studies have demonstrated that various viruses, such as human immunodeficiency virus (35), influenza virus (36), and herpes simplex virus (37), enter the host cells through cholesterol. Additionally, mammalian ApoD is reported to be involved in cholesterol transport (38).

The functional upregulation of ApoD during virus infection was previously unclear. ApoD exerts neuroprotective effects against various neurodegeneration-inducing factors, such as oxidative stress, inflammatory stress, and excitotoxicity, as well as against aging (16, 17, 39, 40). Previously, we reported that ApoD is upregulated in the RABV-infected mouse brain using iTRAQ (**Figure 1A**). Based on this previous iTRAQ data, this study demonstrated that ApoD is upregulated in the mouse brain during RABV infection, as well as in RABV-infected cells *in vitro* (**Figure 1E**). Therefore, this study aimed to examine the role of ApoD during RABV infection and the correlation between ApoD and RABV proteins.

In this study, ApoD overexpression promoted RABV replication in C8-D1A cells. However, this finding is in contrast to the previously reported neuroprotective effects of ApoD. Further analysis revealed that ApoD facilitates RABV replication, which was consistent with the results of a previous study demonstrating that ApoD facilitates the proliferation of *Bombyx mori* nuclear polyhedrosis virus (41).

The G protein forms spikes outside the lipid envelope, recognizes cell receptors through direct interaction, and mediates cell membrane fusion with the M protein to allow RABV to enter the cells. This study investigated the correlation between ApoD and RABV proteins. ApoD could interact with RABV G protein. Additionally, ApoD interacted with G protein from the attenuated strain rRC-HL and the street strain GX074. This indicated that ApoD is a conserved target for RABV G protein, revealing novel biological characteristics (**Figure 4**).

The disruption of cholesterol/sphingomyelin biosynthesis can impair RABV budding (20). This study examined which step of the RABV lifecycle was affected by the interaction. N2A cells were co-transfected with pcDNA-ApoD^-MYC^ and pC-GX074-G^-FLAG^, followed by infection with rRC-HL. The viral titer in the culture supernatant of pcDNA-ApoD^-MYC^/pC-GX074-G^-FLAG^–transfected cells was 2.7-fold and 50-fold higher than that in pcDNA-ApoD^-MYC^–transfected cells at 24 and 48 hpi, respectively (**Figure 5**). Additionally, ApoD–G protein interaction promoted cholesterol synthesis, so we speculate that the ApoD–G protein interaction may probably promote RABV budding. However, further studies are needed to confirm this speculation.

In summary, ApoD was upregulated in the mouse brain (*in vivo*) and C8-D1A cells (*in vitro*) after RABV infection. ApoD overexpression promoted RABV replication in C8-D1A cells. Meanwhile, ApoD–G protein interaction promoted RABV replication by upregulating the cholesterol level. These findings are important to reveal a novel role of ApoD in promoting typical neurotropic virus replication and may provide a potential therapeutic target for rabies.

## Data availability statement

The original contributions presented in the study are included in the article/**Supplementary Material**. Further inquiries can be directed to the corresponding authors.

## Ethics statement

The animal study protocol was approved by the Animal Experiment Committee of Guangxi University with the approval number GXU2019-021. The studies were conducted in accordance with the local legislation and institutional requirements. Written informed consent was obtained from the owners for the participation of their animals in this study.

## Author contributions

HYanZ: Conceptualization, Data curation, Formal analysis, Methodology, Software, Writing – original draft, Investigation, Writing – review & editing. XL: Data curation, Formal analysis, Writing – review & editing. DL: Data curation, Formal analysis, Writing – review & editing. CZ: Software, Writing – review & editing. WW: Software, Writing – review & editing. RT: Data curation, Writing – review & editing. HYunZ: Methodology, Writing – review & editing. ABK: Software, Writing – review & editing. CL: Writing – review & editing, Software. JL: Writing – review & editing, Software. XNL: Conceptualization, Methodology, Supervision, Writing – review & editing. TRL: Conceptualization, Funding acquisition, Methodology, Project administration, Resources, Supervision, Validation, Visualization, Writing – review & editing.

## Supplementary material

Supplementary Table 1 Primers used for quantitative real-time polymerase chain reaction (qRT-PCR) analysis.

Supplementary Figure 1 Apolipoprotein D (ApoD) does not significantly influence the expression of rabies virus (RABV) N, P, and M proteins. **(A–F)** BSR/T7-9 cells were transfected with 1 µg of pcDNA-ApoD^-MYC^ plasmid. At 12 h post-transfection (hpt), 1 µg of pcDNA-rRC-HL-N^-FLAG^, pcDNA-GX074-N^-FLAG^, or pcDNA-CVS-11-N^-FLAG^ was transfected. The cell lysates were collected at 12, 24, and 48 hpt to determine the protein expression levels of rRC-HL N **(A)**, GX074 N **(C)**, and CVS-11 N **(E)** using Western blotting analysis. ImageJ software was used to quantify the rRC-HL N **(B)**, GX074 N **(D)**, and CVS-11 N **(F)** protein levels. The expression levels of N protein were standardized to those of β-actin and normalized to those in cells not transfected with pcDNA-ApoD^-MYC^. **(G–L)** BSR/T7-9 cells were transfected with 1 µg of pcDNA-ApoD^-MYC^ plasmid. At 12 hpt, 1 µg of pcDNA-rRC-HL-P^-FLAG^, pcDNA-GX074-P^-FLAG^, or pcDNA-CVS-11-P^-FLAG^ was transfected. The cell lysates were collected at 12, 24, and 48 hpt to determine the protein expression levels of rRC-HL P **(G)**, GX074 P **(I)**, and CVS-11 P **(K)** using Western blotting analysis. ImageJ software was used to quantify rRC-HL P **(H),** GX074 P **(J)**, and CVS-11 P **(L)** protein levels. The expression levels of P protein were standardized to those of β-actin and normalized to those in cells not transfected with pcDNA-ApoD^-MYC^. **(M–R)** BSR/T7-9 cells were transfected with 1 µg of pcDNA-ApoD^-MYC^ plasmid. At 12 hpt, 1 µg of pcDNA-rRC-HL-M^-FLAG^, pcDNA-GX074-M^-FLAG^, or pcDNA-CVS-11-M^-FLAG^ was transfected. The cell lysates were collected at 12, 24, and 48 hpt to determine the protein expression levels of rRC-HL M **(M)**, GX074 M **(O)**, and CVS-11 M **(Q)** using Western blotting analysis. ImageJ software was used to quantify the rRC-HL M **(N)**, GX074 M **(P)**, and CVS-11 M **(R)** protein levels. The expression levels of M protein were standardized to those of β-actin and normalized to those in cells not transfected with pcDNA-ApoD^-MYC^. Data are represented as mean ± standard deviation. Statistical differences were analyzed using two-way analysis of variance (ns, non-significant).
