# Supplementary material for: Apolipoprotein D facilitates rabies virus propagation by interacting with G protein and upregulating cholesterol
Source: Front Immunol. 2024 May 28;15:1392804. doi: 10.3389/fimmu.2024.1392804 (PMC11167634; doi:10.3389/fimmu.2024.1392804)
Supplement: Supplementary Table 1 — Primers used for quantitative real-time polymerase chain reaction (qRT-PCR) analysis. [file Table_1.docx]

***Supplementary material***

**Supplementary Table 1. Primers used for RT-qPCR.**

| Gene | Primers sequence (5’→3’) | |
| --- | --- | --- |
| ApoD | forward | TCACCACAGCCAAAGGACAAA |
|  | reverse | CGTTCTCCATCAGCGAGTAGT |
| β-actin | forward | AGACCTCTATGCCAACACAGT |
|  | reverse | CATCGTACTCCTGCTTGCTGAT |
| rRC-HL-N | forward | GGCATTGGCAGATGATGGAACT |
|  | reverse | GGCTTGATGATTGGAACTGACTGA |
| CVS-11-N | forward | TGCCGCCAAACTTGATCCG |
|  | reverse | CCTATCTCCTTTTCGTGCAA |
| RABV-P | forward | CATAGAAGACAATCAGGCTC |
|  | reverse | CCTGACCTCATTTGTCTGAC |
| RABV-M | forward | TGATTCCAGGGGCCCTCTTG |
|  | reverse | AAGAGACATGTCAGACCA |
| rRC-HL-G | forward | CTGAGACTTATGGACGGAAC |
|  | reverse | CTGAGACGTCTGAAACTCAC |
| CVS-11-G | forward | GGGTTTTTCGTTGTGTTTCG |
|  | reverse | CCGTTCACTTTGATGGCTGA |
